# A Model for Primary Cilium Biogenesis by Polarized Epithelial Cells: Role of the Midbody Remnant and Associated Specialized Membranes

**DOI:** 10.3389/fcell.2020.622918

**Published:** 2021-01-07

**Authors:** Leticia Labat-de-Hoz, Armando Rubio-Ramos, Javier Casares-Arias, Miguel Bernabé-Rubio, Isabel Correas, Miguel A. Alonso

**Affiliations:** ^1^Centro de Biología Molecular “Severo Ochoa”, Consejo Superior de Investigaciones Científicas and Universidad Autónoma de Madrid, Madrid, Spain; ^2^Department of Molecular Biology, Universidad Autónoma de Madrid, Madrid, Spain

**Keywords:** primary cilium, midbody remnant, centrosome, membrane rafts, condensed membranes

## Abstract

Primary cilia are solitary, microtubule-based protrusions surrounded by a ciliary membrane equipped with selected receptors that orchestrate important signaling pathways that control cell growth, differentiation, development and homeostasis. Depending on the cell type, primary cilium assembly takes place intracellularly or at the cell surface. The intracellular route has been the focus of research on primary cilium biogenesis, whereas the route that occurs at the cell surface, which we call the “alternative” route, has been much less thoroughly characterized. In this review, based on recent experimental evidence, we present a model of primary ciliogenesis by the alternative route in which the remnant of the midbody generated upon cytokinesis acquires compact membranes, that are involved in compartmentalization of biological membranes. The midbody remnant delivers part of those membranes to the centrosome in order to assemble the ciliary membrane, thereby licensing primary cilium formation. The midbody remnant's involvement in primary cilium formation, the regulation of its inheritance by the ESCRT machinery, and the assembly of the ciliary membrane from the membranes originally associated with the remnant are discussed in the context of the literature concerning the ciliary membrane, the emerging roles of the midbody remnant, the regulation of cytokinesis, and the role of membrane compartmentalization. We also present a model of cilium emergence during evolution, and summarize the directions for future research.

## The Primary Cilium

The primary cilium is a non-motile microtubule-based membrane protrusion of the cell surface organized around a central scaffold or axoneme that is surrounded by the ciliary membrane. The primary cilium is present in most mammalian cells, typically attains a length of 3–10 μm, and is present as a single copy (Goetz and Anderson, 2010; Ishikawa and Marshall, 2011). Primary cilia have a basal body that contains structures such as transition fibers and basal feet that derive from the distal and subdistal appendages, respectively, present in the mother centriole, from which the basal body originates. Transition fibers are involved in docking the basal body to the plasma membrane, whereas basal feet anchor cytoplasmic microtubules (Vertii et al., 2016a,b). The axoneme consists of a nine-fold symmetrical arrangement of peripheral microtubule doublets derived from the basal body. The ciliary membrane, which is continuous with, but different from, the plasma membrane, harbors a large variety of receptors for cell signaling, including those for the soluble factors involved in cell growth, migration, development and differentiation, and G-protein-coupled receptors (Singla and Reiter, 2006; Gerdes et al., 2009; Ishikawa and Marshall, 2011). Consequently, ciliary dysfunction causes a great variety of disorders in humans (Novarino et al., 2011; Braun and Hildebrandt, 2017).

## Routes of Primary Cilium Biogenesis

Pioneer work established the existence of two sites for primary cilium assembly (Sorokin, 1962, 1968), whose use depends on the position of the centrosome in the cell: near the nucleus, or close to the cell apex [reviewed by Bernabé-Rubio and Alonso (2017)]. In the former case, the primary cilium assembles intracellularly and produces a cilium that is deeply rooted in the cytoplasm in a membrane invagination known as the ciliary pocket, whereas in the latter case, the cilium forms at the plasma membrane and protrudes from the cell surface (Rohatgi and Snell, 2010; Benmerah, 2013). Mesenchymal cells, like fibroblasts, and polarized epithelial cells, such as those in renal tubules, are examples of cells whose cilium is assembled intracellularly or at the cell surface, respectively.

**Figure 1:**
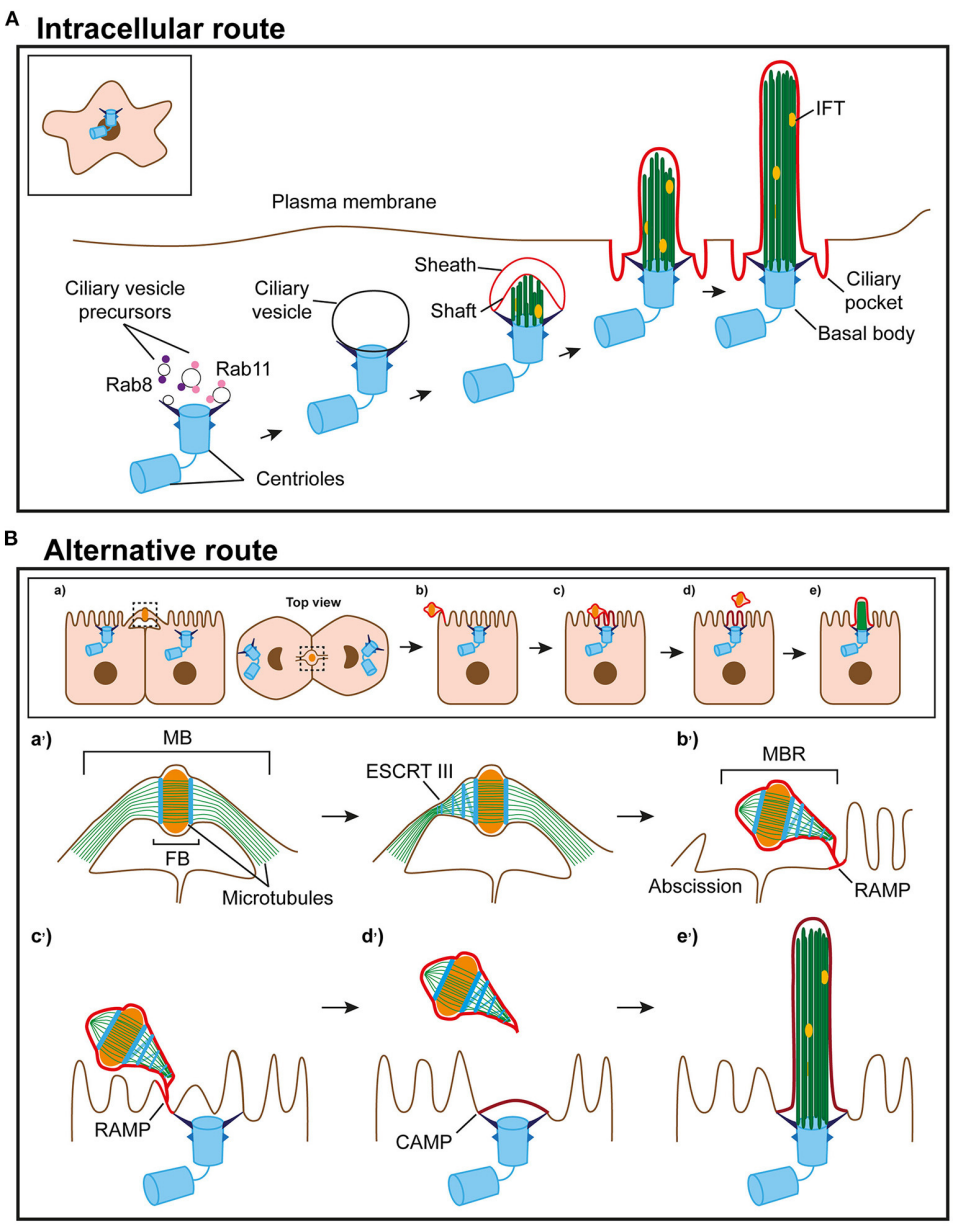
Routes of primary ciliogenesis. **(A)** The intracellular route. Ciliogenesis begins with the formation of a large ciliary vesicle at the distal end of the appendages of the mother centriole by fusion of smaller vesicles. The axoneme starts forming intracellularly and, as it grows, deforms the ciliary vesicle and establishes an inner membrane (shaft) and an outer membrane (sheath). The incipient cilium is finally exocytosed and the cilium becomes exposed in the plasma membrane. The sheath gives rise to the ciliary pocket, and the shaft forms the ciliary membrane. **(B)** Model of the alternative route. (a) In polarized epithelial cells, the intercellular bridge containing ciliary proteins forms at the apical cell surface during cytokinesis. (b) When abscission occurs, the MBR is inherited by one of the daughter cells, acquires a RAMP and localizes at the periphery of the apical surface. (c) The MBR/RAMP moves over the apical surface toward the centrosome, which is docked at the center of the apical membrane. (d) When the MBR is proximal to the centrosome, the RAMP splits into two patches, one of which remains at the MBR and the other, known as the CAMP, occupies the centrosome zone. (e) The ciliary membrane stems from the CAMP. The entire process of primary cilium formation takes place in the plasma membrane. The events occurring at the apical surface in (a–e) are shown in more detail in the corresponding enlargements in (a'-e').

The pathway to intracellular assembly of the cilium begins with the appearance of small vesicles, called primary ciliary vesicles, in the proximity of the older centriole (Sorokin, 1962). These vesicles are transported to the centriole through the sequential action of dynein and myosin MYO5A (Wu et al., 2018). Then, with the participation of the membrane shaping proteins EHD1 and EHD3 (Lu et al., 2015), they fuse to form a larger vesicle, called the ciliary vesicle, that encapsulates the distal end of the mother centriole (Yee and Reiter, 2015). Rab11-Rabin8-Rab8 signaling, Rab23 and Arl13b promote the growth of the ciliary vesicle and the trafficking of ciliary proteins to the growing cilium (Knodler et al., 2010; Westlake et al., 2011; Gotthardt et al., 2015; Gerondopoulos et al., 2019). The two inner microtubules from each of the nine triplets of the centriole gradually elongate and deform the ciliary vesicle, establishing an outer membrane or sheath and an inner membrane or shaft. The machinery for intraflagellar transport (IFT) (Taschner and Lorentzen, 2016) further elongates the incipient axoneme, the ciliary vesicle fuses with the plasma membrane, and the basal body docks with the plasma membrane through the transition fibers, which are derived from the distal centriolar appendages. Upon fusion, the shaft gives rise to the ciliary membrane and the sheath produces the ciliary pocket, and the nascent cilium is exposed to the extracellular space, where it elongates to attain its final length (Sorokin, 1962) (Figure 1A).

In polarized epithelial cells, such as those in the kidney or the monociliated cells of the lung, almost the entire length of the primary cilium is positioned so that it protrudes from the cell surface. A ciliary vesicle also forms in this type of cell, but, unlike in fibroblasts, the ciliary vesicle does not elongate to form an intracellular cilium (Wu et al., 2018), and the cilium assembles at the cell surface (Sorokin, 1962). Multiciliated cells, such as those present in the nose and brain ventricles, extend 30–300 motile cilia from their apical surface (Meunier and Azimzadeh, 2016; Spassky and Meunier, 2017). Before multiciliation, the number of centrioles is amplified, centrioles acquire a ciliary vesicle at their distal end, and dock *en masse* with the apical membrane, where the cilium is assembled. In this case, the ciliary vesicle appears to be involved in the docking of the centriole to the plasma membrane rather than in the assembly of the ciliary membrane (Park et al., 2008). Therefore, according to Sorokin (1962, 1968), the process of ciliogenesis starts intracellularly with the docking of vesicles to the mother centriole. However, the ciliary membrane is assembled at different locations, intracellularly or at the cell surface, and using distinct membrane precursors, the ciliary vesicle or the plasma membrane zone above the docked centrosome, respectively.

Before the work of Sorokin (1962, 1968), Sotelo and Trujillo-Cenoz (1958) observed that centrioles in the neural epithelia of chick embryos attach to the plasma membrane, which then bulges toward the lumen, forming a ciliary bud that is continuous with the plasma membrane. It is of note that no ciliary bud was detected unless the centriole was attached to the plasma membrane. The bud, which initially seems to lack microtubules but contains vesicular structures, appears first to undergo an inward movement toward the nucleus pulled by the basal body, and then an outward movement that exposes the cilium to the extracellular milieu. This mechanism, which was questioned by Sorokin (1968), has not been investigated further but could constitute a route of primary ciliogenesis in which, similar to what was proposed by Sorokin (1968), the ciliary membrane is assembled using the plasma membrane zone above the docked centrosome.

In a large proportion of renal epithelial IMCD3 cells, the centrioles contain ciliary vesicles during the early stages of ciliogenesis but, consistent with Sorokin's model, the cilium is assembled at the plasma membrane (Wu et al., 2018). Renal epithelial MDCK cells also assemble the cilium at the cell surface, but there is no quantitative information about the existence of ciliary vesicles in these cells (Bernabé-Rubio et al., 2016). Throughout this manuscript, we will refer to the route of primary cilium assembly described for fibroblasts as “intracellular,” and as “alternative” when the cilium forms at the cell surface regardless of the presence of ciliary vesicles. Despite its fundamental relevance, research on primary ciliogenesis has concerned itself almost exclusively with the intracellular pathway, whereas the source of membranes for building a primary cilium by the alternative route has remained largely unexplored.

## The Alternative Pathway

### The Midbody Remnant Concentrates Ciliary Machinery and Prepares the Centrosome for Primary Cilium Formation

In animal cells, cytokinesis, the process leading to the physical separation of the daughter cells, begins at anaphase with the assembly of an actomyosin-based contractile ring, known as the cleavage furrow, that progressively constricts the cytoplasm, and the formation of the central spindle, which is a complex structure containing interdigitate antiparallel microtubule bundles, molecular motors and microtubule-associated proteins (Glotzer, 2009). The ingression of the cleavage furrow to the limit results in the formation of the midbody (MB), which is the narrow bridge connecting the two nascent daughter cells before separation. The MB consists of two arms, which contain parallel microtubule bundles, vesicles and protein factors, that flank a 1.0–1.5-μm electron-dense central region, called the Flemming body (FB), which is characterized by densely packed, overlapping antiparallel microtubule bundles emerging from the constricted central spindle (Green et al., 2012). The MB is surrounded by a membrane that is continuous with the plasma membrane. Severing of the MB membrane causes the physical separation of the two daughter cells in a process named abscission (Mierzwa and Gerlich, 2014). Following abscission, the FB and the remainder of the arms form an MB remnant (MBR) that is either released into the extracellular space, in cases where the MB membrane is cleaved in the two arms, or is inherited by one of the daughter cells, when the cleavage occurs in only one of the arms (Chen et al., 2012; Peterman and Prekeris, 2019).

The MBR in polarized epithelial cells initially positions itself at the periphery of the apical surface, close to the cell junctions, since cleavage furrow ingression is more rapid from the basal than from the apical surface (Reinsch and Karsenti, 1994). It is of note that the IFT subunits IFT20 and IFT88, the small GTPase Rab8 and other ciliary machinery concentrate in the MBR of MDCK cells (Bernabé-Rubio et al., 2016), which are considered a paradigm of polarized epithelial cells (Rodriguez-Boulan et al., 2005). The presence of this machinery in the MBR is consistent with the identification of IFT proteins in the cleavage furrow of *Chlamydomonas* (Wood et al., 2012), the existence of cilia-independent functions of ciliary machinery (Vertii et al., 2015), such as the involvement of IFT88 in mitotic spindle orientation (Delaval et al., 2011), and with the results of proteomic analyses of MBs and primary cilia (Skop et al., 2004; Ishikawa et al., 2012; Kohli et al., 2017; Capalbo et al., 2019; Addi et al., 2020), which show a wide spectrum of shared components (Smith et al., 2011).

When MDCK cells proliferate, space becomes limited and cells are progressively constrained by their neighbors. Under these conditions, cells reduce their area of attachment to the substrate, grow in height, establish tight junctions, and form tight cell monolayers with well-differentiated apical and basolateral plasma membrane subdomains (Puliafito et al., 2012). When this succession of events occurs, the MBR transits from its original position at the cell periphery to the center of the apical membrane, which implies that its movement is coupled to the process of cell polarization. MBR movement requires the expression of Rab8 (Bernabé-Rubio et al., 2016). The possibility that Rab8 directly controls the cytoskeleton for MBR movement cannot be ruled out. However, since the best known function of Rab8 relates to membrane trafficking (Peränen, 2011), it is plausible that Rab8 transports materials that facilitate the movement of the MBR or, more probably, that the changes in cell height, formation of junctions, and MBR movement, are different facets of the same program of cell polarization controlled by Rab8.

When the MBR becomes proximal to the centrosome, which is already docked to the center of the apical membrane by the transition fibers, the centrosome starts assembling a primary cilium and the MBR is shed into the extracellular space (Bernabé-Rubio et al., 2016). This collection of sequential events, together with the observation that the physical removal of the MBR greatly impairs primary cilium formation, suggests that the MBR enables the centrosome for primary cilium assembly (Bernabé-Rubio et al., 2016). Therefore, the use of a product from the final stage of cytokinesis, as is the MBR, to license primary ciliogenesis reveals a new connection between cell division and ciliogenesis.

### The MBR Feeds the Centrosome With Specialized Membranes to Build the Ciliary Membrane

Until recently, almost nothing was known about the source of material with which the ciliary membrane is assembled by the alternative route. Using the fluorescent membrane probe Laurdan, whose fluorescence-emission peak depends on the compactness of the lipid environment, it was established that the ciliary membrane of MDCK cells (Vieira et al., 2006) and the flagellum of *Trypanosoma brucei* (Tyler et al., 2009) are enriched in condensed membranes. Compact membranes, sometimes referred to as membrane rafts, have a highly-ordered lipid structure and are involved in the compartmentalization of biological membranes (Simons and Gerl, 2010; Simons and Sampaio, 2011). Lipid liquid-liquid immiscibility is on the basis of the formation of these specialized lipid environments (Dietrich et al., 2001; Bernardino de la Serna et al., 2004; Veatch and Keller, 2005). Using two different environment-sensitive membrane probes, it was observed that peripheral MBRs are surrounded by a patch of compact membranes, which was named the remnant-associated membrane patch (RAMP) (Bernabé-Rubio et al., 2021). The RAMP is acquired by the MBR soon after completion of cytokinesis, and transits together with the MBR from the periphery to the center of the apical surface (Bernabé-Rubio et al., 2021). Although the source of the material of the RAMP is not clear, it could be supplied by vesicles present in the MB before abscission (Fielding et al., 2005; Kouranti et al., 2006; Goss and Toomre, 2008; Schiel et al., 2012). It is of particular note that the RAMP has a degree of membrane condensation, lipid diffusion and molecular lateral mobility similar to that of the ciliary membrane, consistent with a precursor-product relationship between the two membranes (Bernabé-Rubio et al., 2021). A significant observation in living cells was that, once the MBR is in the proximity of the centrosome at the center of the apical surface, the RAMP appears to split into two patches, one of which maintains its association with the MBR, and the other, called the centrosome-associated membrane patch (CAMP), localizes to the plasma membrane zone above the centrosome. Subsequently, the MBR with the rest of the RAMP is shed into the extracellular milieu, and the centrosome starts assembling a primary cilium using the CAMP lipids to build the ciliary membrane (Bernabé-Rubio et al., 2021). Since MDCK cells shed the MBR into the extracellular space once the MBR has prepared the centrosome for primary cilium formation, release of the MBR by epithelial cells, such as those of the neural tube, is not an impediment to the use of the MBR-dependent route, providing that the MBR has fulfilled its function before it is shed.

The sequential precursor-product relationship between the RAMP, the CAMP and the ciliary membrane is supported by different lines of evidence, such as: (a) the physical and genetic removal of peripheral MBRs (Bernabé-Rubio et al., 2016, 2021; Casares-Arias et al., 2020); (b) lipid-tracking experiments (Bernabé-Rubio et al., 2021); and (c) direct monitoring of the process of the assembly of the ciliary membrane in live cells (Bernabé-Rubio et al., 2021). MAL, which is an integral membrane protein that has been involved in condensed membrane organization (Puertollano et al., 1999; Antón et al., 2008; Magal et al., 2009), is necessary for proper membrane condensation at the ciliary base (Reales et al., 2015), and could act jointly with other machinery to maintain the condensation of the RAMP/CAMP. In summary, we envision the alternative route as a sequential process by which the MBR acquires condensed membranes soon after the end of cytokinesis, moves along the apical surface to a central position, and feeds the centrosome with condensed membranes for ciliary membrane assembly (Figure 1B).

### Regulation of MBR Inheritance

**Figure 2:**
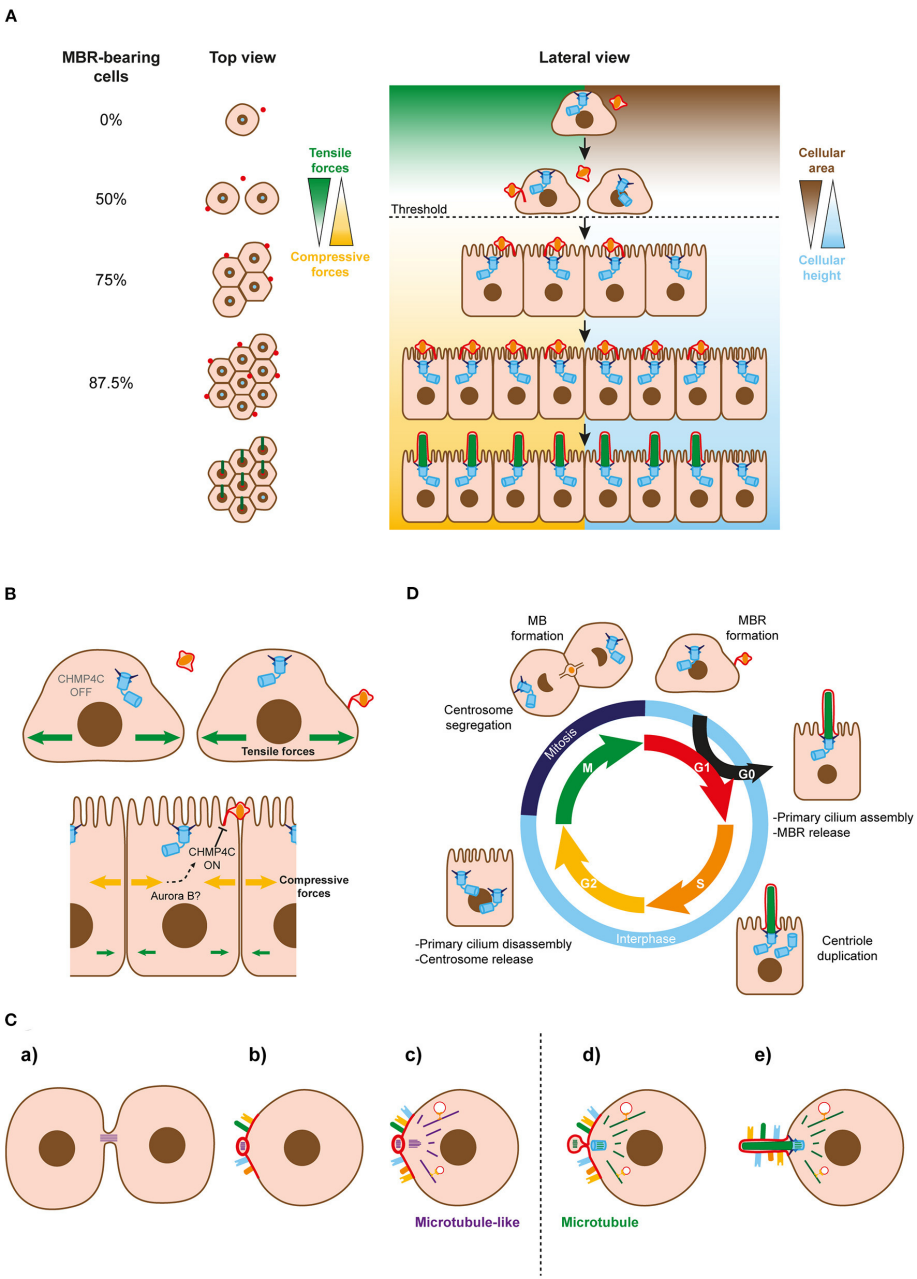
Regulation of MBR inheritance at the cell-population and single-cell levels. **(A)** Simplified model of MBR retention at the cell-population level. When the area of the cells falls below a threshold (discontinuous line), cells continue dividing and generating new midbody remnants that move to the cell center and enable ciliogenesis. Successive cycles of cell division increase the number of MBR-bearing cells exponentially. The remaining fraction of cells without an MBR could be kept to generate a small pool of non-ciliated cells or be extruded from the cell monolayer. **(B)** Role of CHPM4C in MBR retention. A membranous stalk physically connects the MBR membrane and the plasma membranes of most MBR-containing cells. The stalk is derived from the uncleaved arm of the bridge and contains ESCRT machinery, including the regulatory subunit CHMP4C. CHMP4C acts on the second cut of the bridge membrane to preserve the MBR membrane connected to the plasma membrane. The integrity of the connection is necessary at the single-cell level to enable the centrosome to form a primary cilium. The change from tensile to compressive forces might act on CHMP4C in some way to prevent the cleavage of the second MB arm, allowing the connected MBRs to be inherited. **(C)** Schematics of the proposed evolutionary process leading to cilium emergence. (a) The intercellular bridge acquired microtubule-like filaments during prokaryotic-to-eukaryotic cell evolution. A primitive remnant containing this type of filament originated after the daughter cells separate. (b) The membrane of the remnant progressively acquired receptors and specialized lipids and formed a sensory patch. (c) A primordial microtubule-like organizing center emerged near the patch to nucleate microtubule-like filaments that, with the emergence of molecular motors, served as tracks for vectorial transport to the patch. (d) Microtubules replaced the microtubule-like filaments. The MTOC and the bridge remnant co-evolved in a basal body and an MBR, respectively. (e) The basal body acted as template for the formation of the axoneme, and the patch became the ciliary membrane. Since it was no longer necessary for it to remain at the plasma membrane, the MBR was discarded after the cilium had been assembled. At the beginning of the evolutionary process, both daughter cells probably inherited part of the bridge as a remnant. The acquisition of ESCRT machinery later on during evolution enabled MBR inheritance regulation. The discontinuous line indicates the transition between microtubule-like filaments and microtubules. **(D)** The three microtubule-based organelles and the cell cycle. The centrosome functions as a basal body in the cilium when cells are in the G0 phase, and is repurposed as the major MTOC when the cilium is disassembled and the cell returns to the cell cycle. It distributes to the poles of the mitotic spindle during the M phase. Once mitosis ends, with the formation of two identical nuclei, the MB forms to divide the cytoplasm into two halves, and gives rise to the MBR when the process of abscission is completed. Cells enter the G1 phase with the centrosome and the MBR in readiness to become engaged in primary cilium assembly.

Our model of primary ciliogenesis implies that only cells with an MBR can become ciliated. Since only one MBR forms after cell division, it may seem at first glance that, at best, only 50% of cells can ciliate, raising the question as to how a population of cells, for instance in a tissue, can come to comprise a higher percentage of ciliated cells. To resolve this apparent paradox, a model of primary ciliogenesis at the cell-population level was developed, based on mathematical simulations that were supported by quantitative data and videomicroscopic analyses (Bernabé-Rubio et al., 2016). MBR conservation and the beginning of primary ciliogenesis appear to be controlled in a cell-confinement-dependent manner whose threshold marks the transition from a rapid to a slow regime of cell division. Since not all the cells in the population behave synchronously, the model is more complex than the one depicted (Figure 2A) but, essentially, proposes that, just before becoming competent for forming a primary cilium, the cell that has reached the threshold maintains the MBR and divides in such a way that one of the daughter cells receives a new MBR and the other maintains the old one. In this way, the number of MBR-bearing cells grows exponentially and, after a few cycles of cell division, most cells will have one MBR and, consequently, the potential to form a primary cilium. Therefore, MBR retention is important not only at the single-cell level, in order to provide membranes to the centrosome for primary cilium assembly, but also at the cell-population level, to guarantee a high percentage of MBR-bearing cells.

The final steps of the abscission process are carried out by the endosomal sorting complexes required for transport (ESCRT) III machinery (Carlton and Martin-Serrano, 2007; Morita et al., 2007; Schöneberg et al., 2017), which progressively accumulates into rings on both sides of the FB (Elia et al., 2011; Casares-Arias et al., 2020). Afterwards, but before membrane cleavage, the ESCRT-III assembles spiral polymers whose diameter decreases as they grow away from the FB, constricting the MB (Goliand et al., 2018). The first cleavage of the MB membrane, which is sufficient to separate the daughter cells, is regulated by the regulatory ESCRT III subunit charged multivesicular body protein (CHMP) 4C, through the abscission checkpoint mechanism. This mechanism retards abscission in the case of mitotic problems, and requires phosphorylation of CHMP4C by the kinase Aurora B (Capalbo et al., 2012; Carlton et al., 2012). Ultrastructural analyses by transmission and scanning electron microscopies indicate that the membrane of most MBRs of MDCK cells is connected to the adjacent plasma membrane by a membranous stalk (Bernabé-Rubio et al., 2016; Casares-Arias et al., 2020). Detailed analysis of the cytokinetic process revealed that only one of the two MB arms is cleaved for abscission in most cases, and that the stalk derives from the arm that remains uncleaved. The integrity of the stalk is regulated by CHMP4C (Casares-Arias et al., 2020) (Figure 2B). Ser/Thr residues that are phosphorylated when the abscission checkpoint mechanism is activated, and that are crucial for retarding the first cut of the MB membrane, are also critical for delaying the second cleavage (Casares-Arias et al., 2020). When cells proliferate and the availability of space becomes limited, compressive stress forces replace those of tensile stress (Trepat et al., 2009; Bazellières et al., 2015). Since MBR inheritance in MDCK cells is regulated by cell confinement, we propose that the change in stress forces is somehow transmitted to CHMP4C to prevent the cleavage of the second MB arm, thereby allowing the inheritance of connected MBRs (Figure 2B).

In the absence of CHMP4C, the connection is cleaved and, consequently, the MBR is shed into the extracellular space. This dramatically reduces the percentage of ciliated cells (Casares-Arias et al., 2020), supporting the results obtained by physical removal of the MBR (Bernabé-Rubio et al., 2016, 2021), and providing genetic evidence of the requirement for MBR in primary cilium formation. The membranous connection ensures physical continuity between the MBR and the plasma membrane, and allows the transit of condensed membranes, and probably of other ciliary components, from the MBR to the plasma membrane zone where the centrosome is docked.

The ESCRT-associated VPS4 protein and five ESCRT subunits were isolated from *Chlamydomonas* flagella (Diener et al., 2015). Although their function was not investigated, they might be associated with the budding of vesicles (ectosomes) from the flagella, the process of flagellum formation, and the transport of ubiquitinated ciliary proteins from the flagellum base to multivesicular endosomes for degradation during flagellar reabsorption. VPS4 was also found at the centrosome of NIH-3T3 fibroblasts (Ott et al., 2018). The expression of a dominant-negative form of VPS4 reduces primary cilium formation in these cells and also in zebrafish embryos. Under these conditions, ~30% of NIH-3T3 cells have a large vesicle at the distal end of their mother centriole, suggesting that the VPS4 impedes the progress of the process of ciliogenesis beyond this stage (Ott et al., 2018). The ESCRT-III components CHMP2A and CHMP4A are at the base and along the cilium, respectively, of RPE1 and IMCD3 cells (Jung et al., 2020). CHMP4B depletion reduces the number of ciliated cells, indicating that CHMP4B is necessary for proper primary cilium formation (Jung et al., 2020). Thus, it is clear that ESCRT and ESCRT-associated proteins may have multiple roles that could affect ciliogenesis. However, for the CHMP4C subunit, the only effect on ciliogenesis known so far is its role in regulating MBR inheritance (Casares-Arias et al., 2020).

### Proteins and Lipids at the MBR and the Ciliary Membrane

The proteome of the MB contains ciliary machinery and a large number of signaling proteins (Skop et al., 2004; Capalbo et al., 2019; Addi et al., 2020). Rab8 and IFT components were found at the MBR (Bernabé-Rubio et al., 2016), but the protein content of the CAMP is not known. Therefore, it is plausible that some ciliary proteins are provided together with CAMP lipids, although most ciliary proteins are known to be delivered to the ciliary base by vesicular transport (Sung and Leroux, 2013), and enter the cilium through the ciliary gate, which is the region at the ciliary base separating the cilium from the cytoplasm (Garcia-Gonzalo and Reiter, 2017). The material supplied by the MBR appears to be enough to build a normal-sized cilium (Bernabé-Rubio et al., 2021). Once it is formed, the cilium can be elongated by using membranes delivered by vesicular transport, as occurs after disruption of the actin cytoskeleton (Kim et al., 2010). In these cases, the increased ciliary length seems to be due to an extra supply of membranes to the centrosome that is facilitated by the clearance of actin filaments from the centrosome zone (Kim et al., 2010). A similar increase in vesicular delivery to the centrosome is observed in MDCK cells in which the apical actin cytoskeleton was disrupted (Rangel et al., 2019).

Lipids from the CAMP are delivered by the MBR to the centrosome and are subsequently used to assemble the ciliary membrane (Bernabé-Rubio et al., 2021). The discovery that RAMPs are formed by specialized membranes is consistent with those of previous studies showing that cells specifically regulate the localization of lipids to the MB (Atilla-Gokcumen et al., 2014), that lipids characteristic of condensed membranes are required for cytokinesis (Abe et al., 2012; Makino et al., 2015), and that the MB has a different lipid composition from that of most cellular membranes (Arai et al., 2015). Phosphorylated derivatives of phosphatidylinositol (PI) modulate many cellular processes, such as membrane trafficking, cell signaling and cytoskeleton organization (Di Paolo and De Camilli, 2006; Cauvin and Echard, 2015) and are important for cytokinesis and normal function of the cilium (Kouranti et al., 2006; Logan and Mandato, 2006; Garcia-Gonzalo et al., 2015; Phua et al., 2017). PI(4,5)P_2_ interacts directly with several actin-binding proteins to activate the assembly of actin filaments and to inhibit their disassembly (Saarikangas et al., 2010). PI(4,5)P_2_ hydrolysis is catalyzed by PI5 phosphatases, such as OCRL and Inpp5e, which generate PI(4)P. Inpp5e, which localizes to the cilium, compartmentalizes PI(4)P to the ciliary membrane and PI(4,5)P_2_ to the ciliary base (Garcia-Gonzalo et al., 2015). This segregation prevents actin filament formation in the cilium and is necessary for normal transport of GPCRs to the cilium, for ciliary signaling and cilium stability (Garcia-Gonzalo et al., 2015; Phua et al., 2017). OCRL, which is transported to the intercellular bridge during cytokinesis, is responsible for PI(4,5)P_2_ hydrolysis, allowing actin filament clearance, which is a necessary step for normal cleavage of the MB membrane (Kouranti et al., 2006; Logan and Mandato, 2006). Although there is no direct information about the occurrence of PIs in the MBR, PI(4)P might be present there, since PI(4)P is generated just before MBR formation.

## Speculative Model of Cilium Emergence During Evolution

It was suggested that the evolutionary origin of the cilium was a specialized membrane patch of the plasma membrane that recruited important sensory receptors. During evolution, the primitive patch progresses by acquiring microtubules to make it protrude for increased environmental exposure of sensory membranes, and also by the appearance of a primeval machinery derived from a coatomer-like progenitor for vectorial transport to the membrane patch (van Dam et al., 2013). Subsequently, the scaffold microtubules and the patch become the axoneme and the ciliary membrane patch, respectively, the primitive machinery evolve to become the IFT machinery, and cilia are also used for cell propulsion (Jékely and Arendt, 2006; Quarmby and Leroux, 2010; Bloodgood, 2012).

In eukaryotes, chromosome segregation is performed by the tubulin-based cytoskeleton, whereas cytokinesis involves the actin–myosin cytoskeleton. In contrast, most prokaryotic cytokinesis is based on the tubulin homolog FtsZ, whereas actin-like proteins, such as MrB, are used for chromosome segregation. This suggests that a profound switch in the mechanism of cell division has occurred during the transition from prokaryotes to eukaryotes (Löwe and Amos, 2009; Jékely, 2014). We speculate that after this switch, the primitive intercellular bridge with microtubule-like filaments generates a remnant that progressively acquires specialized lipids to assemble a membrane patch that serves to recruit sensory receptors, and develops some of the ciliary functions before the cilium emerges. Although the centrosome may have originated independently of the ancient bridge remnant, it is also plausible that the microtubule-like filaments of a primitive remnant give raise autogenously to a primordial microtubule-organizing center (MTOC) close to the patch, whose original mission might be to direct vectorial transport of additional receptors or new materials to the patch. The primitive intercellular bridge and MTOC could have co-evolved thereafter. On one hand, the bridge replaces microtubule-like filaments with microtubules, forms an FB, become an MB and, upon abscission, an MBR. On the other hand, the MTOC might become a basal body capable of acting as a template for the formation of a cilium and, afterwards, a centrosome, by duplication and acquisition of a periocentriolar matrix (Figure 2C). This proposed evolutionary pathway resembles the model of primary cilium biogenesis that we have presented for the alternative route of polarized epithelial cells (Figure 1B).

## Conclusions and Future Directions

The bipartite, functional links between the centrosome and cell division, and therefore between the centrosome and the MB/MBR, and between the centrosome and the cilium are well-established. More than 125 years after the discovery of the centrosome, the MB and the cilium, our model of primary ciliogenesis establishes a tripartite link between these microtubule-based organelles by revealing that the MBR feeds the centrosome with specialized membranes forming an immiscible lipid liquid-liquid patch to assemble the primary cilium.

One important question is whether, in addition to transferring the patch of condensed membranes, the MBRs delivers other materials to the cell that inherits it (Peterman et al., 2019). Other questions address the movement of the MBR to meet the centrosome. How is the MBR propelled to transit to the center of the apical surface? How does it communicate with the centrosome to know where to stop? It is plausible that the use of MBR material for ciliogenesis is not exclusive to the alternative route. It will be worthwhile investigating whether the MBR, either at the plasma membrane or internally, participates in the intracellular route by providing material to form the small vesicle precursors.

Our model of primary ciliogenesis by the alternative route is closely linked to cell division (Figure 2D). It proposes that a part of the MB, in the form of the MBR, is repurposed after cell division to deliver specialized membranes to the centrosome for the assembly of the ciliary membrane. The model is based entirely on experimental results and prompts new questions that need to be addressed. To make progress in this area, it is necessary to put aside dogmatic positions and evaluate the model with new experiments. The words of Sorokin (1962), concerning his model of the intracellular route, are equally relevant to our model of the alternative route: “*there is some reason to remark that the reconstruction of a biological process from a series of stages can express a hypothesis, but that it does not establish its truth //…//. Some merit may therefore be found in the reconstructions offered if they are helpful in establishing the conceptual framework that so often precedes the design of subtle and telling experiments*.”

## Author Contributions

LL-d-H: prepared the first draft and made the figures. AR-R: collaborated in the preparation of the draft. JC-A and MB-R: corrected the draft. IC: supervised the writing of the manuscript. MA: designed the work and supervised the writing of the manuscript. All authors contributed to the article and approved the submitted version.

## Conflict of Interest

The authors declare that the research was conducted in the absence of any commercial or financial relationships that could be construed as a potential conflict of interest. The handling editor declared a shared affiliation with one of the authors IC.

## Abbreviations

CAMP: centrosome-associated membrane patch
CHMP: charged multivesicular body protein
ESCRT: endosomal sorting complexes required for transport
IFT: intraflagellar transport
FB: Flemming body
MB: midbody
MBR: MB remnant
RAMP: remnant-associated membrane patch.
